# Pharmacist-Led Diagnostics: A New Frontier in Antimicrobial Stewardship

**DOI:** 10.3390/antibiotics14121286

**Published:** 2025-12-18

**Authors:** Greta Kaspute, Tatjana Ivaskiene

**Affiliations:** State Research Institute Centre for Innovative Medicine, Santariskiu St. 5, LT-08410 Vilnius, Lithuania; tatjana.ivaskiene@imcentras.lt

**Keywords:** pharmacists, point-of-care testing, antibacterial agents, antibiotics, antimicrobial resistance

## Abstract

**Background:** Antimicrobial resistance (AMR) is accelerating globally, driven by widespread inappropriate antibiotic use and diagnostic uncertainty in primary care. Pharmcist-led point-of-care testing (POCTs) has emerged as a promising model to optimize antibiotic prescribing, improve triage, and strengthen antimicrobial stewardship (AMS). **Methods:** This scoping review synthesizes current evidence, regulatory models, and implementation data on pharmacist-led diagnostics and antibiotic management across multiple countries. **Results:** Despite strong policy interest, clinical trial evidence remains limited. Existing studies—primarily pilots, feasibility work, and service evaluations—suggest that POCTs combined with structured pathways reduces inappropriate antibiotic use. **Conclusions:** Regulatory fragmentation, workforce limitations, and insufficient monitoring systems constrain widespread implementation. Larger, well-designed trials are needed to establish long-term safety, clinical outcomes, and AMS impact.

## 1. Introduction

Bacterial antimicrobial resistance (AMR) occurs when bacteria evolve to withstand the drugs designed to eliminate them, rendering treatments less effective [1]. AMR has emerged as one of the most pressing public health threats of the 21st century, with a critical global burden. In 2019 alone, bacterial AMR was directly responsible for 1.27 million deaths worldwide and contributed to nearly 5 million fatalities [1,2]. In the United Kingdom (UK), 81% of all antibiotic prescriptions originate in primary care and community settings, with up to 20% estimated to be inappropriate [3]. Acute conditions such as sore throat account for a significant portion of these prescriptions, despite the majority being viral in origin and self-limiting [4].

In many low- and middle-income countries (LMICs), including Nigeria, limited access to functioning primary healthcare centers has shifted the responsibility for frontline care to private community pharmacies (PCPs) [5]. These pharmacies, staffed by pharmacists and pharmacy assistants, provide essential primary care services and are well-positioned to influence appropriate antibiotic use. Effective antimicrobial stewardship (AMS) in such settings is critical to prevent antibiotic overuse, reduce drug resistance, decrease healthcare costs, and improve patient outcomes [6].

Point-of-care tests (POCTs) offer a practical approach to address diagnostic uncertainty, particularly in conditions such as respiratory tract infections (RTIs), where inappropriate antibiotic prescribing is common [7]. POCTs performed in community and ambulatory care pharmacies can facilitate screening, diagnosis, treatment decisions, and referral for both acute and chronic conditions. Growing evidence demonstrates that pharmacist-led POCTs, combined with clinical protocols and training, can reduce unnecessary antibiotic use, improve patient care, and support public health objectives [8].

Despite the growing recognition of pharmacists’ potential role in AMS, substantial challenges remain. Developing new antibiotics is scientifically and economically difficult, and the pharmaceutical industry has largely deprioritized antibiotic research. Large-scale pharmaceutical companies have reduced or exited antibiotic R&D, leaving small biotech companies and academic institutions as the main contributors. Coupled with a global shortage of antimicrobial researchers and long regulatory timelines, these factors exacerbate the difficulty of responding to AMR [9]. Meanwhile, antibiotic consumption continues to rise globally, further underscoring the urgency of stewardship interventions [1].

Community pharmacists are uniquely positioned to contribute to AMR mitigation. They are accessible healthcare providers who can perform rapid diagnostic tests, offer clinical advice, and in some jurisdictions, prescribe antibiotics. With appropriate regulatory support, POCTs programmes in community pharmacies represent a promising strategy to optimize antimicrobial use, enhance patient care, and strengthen public health initiatives [10].

The aim of this article is to raise awareness of the current evidence, global regulatory landscape, challenges, and future opportunities for pharmacist-led interventions in antimicrobial stewardship.

## 2. Results

### 2.1. Regulation by Country

#### 2.1.1. United States

In the **United States (U.S.)**, pharmacists’ ability to prescribe or initiate antibiotic therapy following POCTs remains inconsistent and is largely dictated by state laws, board of pharmacy regulations, and enabling statutes [11]. In most states, pharmacists lack independent prescribing authority unless it is explicitly granted or delegated through regulation [12].

Some states allow pharmacists to practice under collaborative practice agreements (CPAs) or physician-approved standing protocols. These arrangements authorize pharmacists to perform certain diagnostic tests and, under defined conditions, initiate antibiotic therapy according to pre-set algorithms [13]. More recently, several states have adopted “test-and-treat” legislation or broader scope-of-practice laws that permit pharmacists to conduct rapid testing for influenza, COVID-19, and group A *Streptococcus*, and in some cases prescribe treatment based on results, though always within regulated boundaries [14,15]. Overall, the regulatory environment is highly state-dependent: many jurisdictions continue to prohibit independent prescribing, while others permit only limited test-and-treat services.

Across both U.S. and international models, pharmacists’ prescribing authority is most often applied to a small set of clinical conditions and antibiotic classes [16]:

**Streptococcal pharyngitis (strep throat)**: Pharmacists use rapid antigen detection tests (RADTs) for group A *Streptococcus*. If positive and the patient meets eligibility criteria (e.g., no high-risk features or complications), pharmacists can dispense or prescribe first-line antibiotics such as penicillin or amoxicillin [15]. Evidence shows pharmacist-led RADT programs reduce inappropriate prescribing, with antibiotic use rates of ~24–26% compared to historically higher levels [17].

**Uncomplicated urinary tract infections (UTIs)**: Some protocols allow pharmacists to manage low-risk, healthy women with uncomplicated UTIs using symptom-based assessment, sometimes supported by urine dipstick testing. Recommended antibiotics typically include short courses of nitrofurantoin or trimethoprim [17].

**Respiratory infections**: Certain pharmacy-based “cough, cold, and flu” services incorporate POCTs for influenza or group A *Streptococcus*. While these programs often emphasize antivirals or symptomatic care, they establish infrastructure that aligns with antimicrobial stewardship efforts [18].

In 2022, U.S. clinicians issued approximately 236.4 million outpatient oral antibiotic prescriptions—equivalent to 709 prescriptions per 1000 people—according to IQVIA Xponent^®^ data. Prescribing patterns varied by age, sex, and region: children and adolescents (<20 years) received 48.5 million prescriptions (598 per 1000), while adults (≥20 years) accounted for 187.6 million (744 per 1000). Women had higher prescribing rates (144.9 million; 862 per 1000) than men (91.3 million; 552 per 1000). Regionally, the South had the highest prescribing rate (106.2 million; 825 per 1000), followed by the Northeast (41.1 million; 721 per 1000) and Midwest (49.5 million; 719 per 1000). The West reported the lowest rate (39.5 million; 502 per 1000) [19,20].

#### 2.1.2. United Kingdom

In the **UK**, pharmacists’ authority to prescribe antibiotics is shaped by national regulations and professional training frameworks rather than state-level variability [21]. Traditionally, pharmacists have supplied antibiotics under Patient Group Directions (PGDs), which allow medicine supply for specific conditions within tightly defined criteria, but do not grant broad independent prescribing rights [22]. More recently, the Pharmacy First initiative in the UK (launched in January 2024) has expanded community pharmacists’ role by enabling them to assess and manage seven common conditions—including sore throat, sinusitis, earache, impetigo, infected insect bites, shingles, and uncomplicated urinary tract infections in women—using structured clinical pathways and PGDs [23].

Alongside this, the expansion of Independent Prescribing training means that from 2026, all newly qualified pharmacists will automatically graduate as independent prescribers, significantly increasing capacity for direct antibiotic prescribing [24]. Evidence from pilot “minor ailments” services shows pharmacists generally adhere to PGD criteria, with only a subset of patients meeting eligibility for antibiotics, highlighting their role in antimicrobial stewardship [25]. National surveillance indicates that overall antibiotic consumption in the UK was 17.6 defined daily doses (DDD) per 1000 inhabitants per day in 2023, lower than pre-pandemic levels, with primary care accounting for around 72% of prescriptions. Despite progress, studies estimate at least 20% of community prescribing remains inappropriate, and a small proportion of patients account for the majority of repeat antibiotic courses [26].

#### 2.1.3. Australia

In **Australia**, the role of community pharmacists in antibiotic prescribing is expanding through pilot programs and policy initiatives aimed at enhancing access to primary care and promoting antimicrobial stewardship. Notably, the Victorian Government’s Community Pharmacist Program, launched in October 2023, allows trained pharmacists to independently prescribe for conditions such as UTIs, shingles, and mild plaque psoriasis without a prior general practice (GP) prescription [27].

Similarly, a national pilot study conducted in 2024 assessed community pharmacists’ referrals for suspected antibiotic-requiring infections. Out of 466 minor ailment encounters, 16.5% (77 cases) were referred to general practice, indicating that pharmacists are discerning in their decision-making and adhere to clinical guidelines when considering antibiotic therapy [28].

These initiatives align with Australia’s broader efforts to address high antibiotic prescribing rates. A qualitative study in 2025 highlighted that while community pharmacists are willing to engage in antimicrobial stewardship, challenges such as limited interprofessional communication and the current funding model hinder their full participation. Recommendations include fostering better collaboration with general practitioners and revising funding structures to support pharmacists’ involvement in antimicrobial stewardship [29].

#### 2.1.4. Canada

In **Canada**, provinces such as Alberta and Ontario, pharmacists have been granted the authority to prescribe certain antibiotics for conditions such as uncomplicated UTIs, provided they follow specific guidelines and protocols [30].

Pharmacists in Canada are also involved in POCTs for conditions like strep throat. In British Columbia, for instance, pharmacists can assess and treat 21 minor ailments, including UTIs, allergies, pink eye, and dermatitis [31]. They can prescribe and dispense contraceptives, renew or change some prescriptions, and provide emergency supplies. These services are free for residents when provided in person at a pharmacy. Additionally, the Government of Saskatchewan has launched services allowing pharmacists to provide one-stop testing for strep throat and ear infections, and to prescribe and dispense medication as needed [32].

CPAs further enhance pharmacists’ roles in patient care. These agreements establish formal partnerships between pharmacists and other healthcare providers, allowing pharmacists to manage and modify drug therapy according to specific protocols. For instance, in New Brunswick, pharmacists can participate in CPAs to prescribe medications and treatments as outlined in the agreement.

#### 2.1.5. Brazil

In **Brazil**, the role of community pharmacists in antibiotic prescribing is evolving, primarily through regulatory frameworks and national programs aimed at enhancing access to healthcare and promoting antimicrobial stewardship [33].

While independent prescribing by pharmacists is not standard practice, there have been advancements in expanding their roles. The Federal Pharmacy Council (CFF) implemented Resolution CFF No. 586/2013, allowing pharmacists to prescribe certain medications, such as hormonal contraceptives, provided they adhere to specific protocols and are adequately trained [34].

Additionally, national antimicrobial stewardship programs have been established to promote rational antibiotic use. A study evaluating the implementation of such programs in Brazilian hospitals found that 47.5% of hospitals had active antimicrobial stewardship initiatives, supported by factors like top management commitment and dedicated antimicrobial stewardship teams [35].

Despite these advancements, challenges remain. A study assessing Brazilian community pharmacists’ knowledge of medication dispensing practices revealed that while pharmacists recognize the importance of antimicrobial stewardship, there are gaps in their knowledge and practices [36].

### 2.2. Clinical Data, Monitoring

**Figure 1:**
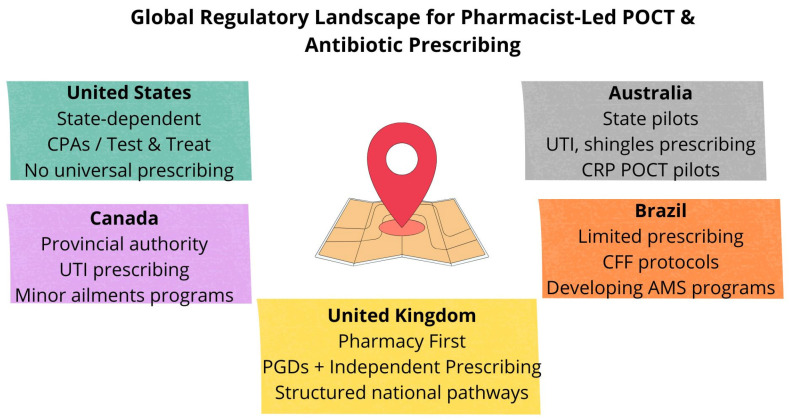
Global Regulatory Landscape for Pharmacist-Led POCTs and Antibiotic Prescribing.

Across countries where pharmacists are permitted to initiate antibiotic therapy after POCTs, clinical data collection and follow-up monitoring remain highly variable. In many U.S. “test and treat” models, pharmacists document test results, prescribing decisions, and patient eligibility criteria within standardized protocols, with data often shared back to physicians under collaborative practice agreements [37]. In the U.K.’s Pharmacy First service, structured clinical pathways and PGDs ensure that test results (e.g., strep throat rapid antigen tests) and antibiotic supplies are recorded electronically, with oversight from NHS commissioning bodies [4,38]. Australian pilot programs, such as those in Victoria and Western Australia, also emphasize careful data capture: pharmacists record patient demographics, test outcomes, and prescribing decisions, with evaluation reports used to assess safety and antimicrobial stewardship outcomes [39,40]. In most models, ongoing monitoring is limited to audit and reporting at the system level rather than routine patient follow-up (Figure 1).

While pharmacists may advise patients to return if symptoms persist or worsen, active post-treatment follow-up (such as phone calls) is generally uncommon outside of research settings. Instead, quality assurance relies on protocol adherence, referral back to general practitioners when exclusion criteria are met, and aggregated data analysis to ensure that antibiotics are prescribed appropriately and only for eligible cases. This framework reflects a balance between increasing access to care and maintaining robust oversight for antimicrobial stewardship.

Clinical data on pharmacist-led point-of-care testing and antibiotic prescribing have been primarily generated through cluster randomized trials, pilot programs, and evaluation studies. A summary of these findings is presented in Table 1.

Among the studies identified in this review, most European research on POCTs and antibiotic prescribing originates from the United Kingdom. Evidence from other European countries is extremely limited, with few, if any, published studies evaluating community pharmacy–based POCTs and its impact on antibiotic dispensing or prescribing. While POCTs is widely implemented in general practice settings across Europe, comparable data for community pharmacies are scarce. This highlights a significant geographic evidence gap, suggesting that pharmacy-led POCTs initiatives are either under-reported or largely confined to pilot or unpublished programs in continental Europe. Expanding the search to include non-English publications or grey literature, such as conference abstracts, national reports, and pharmacy association documents, may help capture additional evidence.

## 3. Discussion

This scoping review highlights rapid growth in interest surrounding pharmacist-led diagnostics while simultaneously exposing a substantial evidence–practice gap. Although the literature base exceeds 382 publications, most are secondary analyses rather than primary clinical evaluations, leaving 48 suitable for review. In addition, health policy in many countries is advancing faster than the availability of rigorous evidence to guide safe implementation. Despite this imbalance, the existing research consistently shows that pharmacist-led POCTs is feasible, acceptable to both pharmacists and patients, and associated with reductions in unnecessary antibiotic use. CRP testing appears particularly valuable for triaging respiratory tract infections, and RADTs enable more targeted treatment of streptococcal pharyngitis. Notably, evidence from Wales and Nigeria demonstrates that POCTs delivered within structured training and protocol frameworks can significantly reduce inappropriate antibiotic supply and ease the burden on primary care.

A major finding of this review is the uneven methodological strength across studies. Although interest in pharmacist-led POCTs is high, the number of rigorously designed randomized trials remains very limited. Most evaluations are feasibility pilots, early implementation studies, or service assessments. As a result, the evidence base is characterized by considerable heterogeneity in test modalities, intervention structures, and outcome measures, making it difficult to synthesize results or establish generalizable conclusions. One notable randomized trial, conducted in Nigeria, demonstrated that CRP testing combined with pharmacist training reduced non-prescription antibiotic dispensing for respiratory infections, although such context-specific evidence remains limited [5]. Furthermore, while high-income countries provide substantial implementation data due to more developed pharmacy infrastructures, findings may not fully translate to lower- and middle-income settings, where patterns of antibiotic misuse and access differ. Conversely, evidence from resource-limited contexts remains too sparse to guide broad policy recommendations.

Another limitation of the current literature is its narrow focus on immediate clinical decisions. Most studies assess short-term outcomes such as dispensing, prescribing, or referral, without examining longer-term clinical recovery, reconsultation, antimicrobial resistance trends, or the economic sustainability of pharmacy-led testing. Microbiological endpoints and cost-effectiveness analyses are almost entirely absent, despite their importance for informing national antimicrobial stewardship strategies. Additionally, little is known about how regulatory environments, funding models, or governance structures influence the feasibility and scalability of POCTs services, yet these system-level factors are likely to be decisive in shaping real-world adoption.

**Figure 2:**
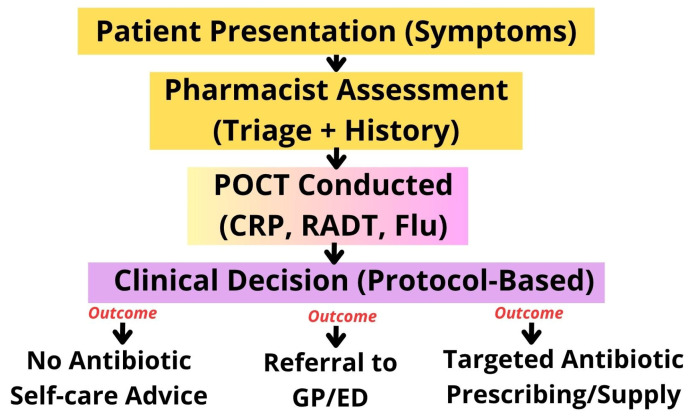
Conceptual Model of Pharmacist-Led Antimicrobial Stewardship (AMS) Pathway.

A further gap relates to antibiotic adherence and household storage of leftover antibiotics, both persistent drivers of misuse. Pharmacists, as accessible medication experts, are well positioned to provide follow-up and adherence support, but no current POCTs model meaningfully incorporates this component (Figure 2). Integrating follow-up mechanisms—whether through structured counselling, digital tools, or repeat contact—could strengthen the stewardship impact of pharmacy-based diagnostics.

Overall, the available evidence suggests that pharmacist-led POCTs can play an important role in antimicrobial stewardship when supported by clear protocols, appropriate training, and alignment with broader primary care systems. However, significant work remains to fully establish its effectiveness, safety, and transferability. Future research should prioritize high-quality randomized and controlled trials that measure both clinical and microbiological outcomes, incorporate longer follow-up, and include economic evaluations. Harmonizing outcome measures across studies would enable more meaningful comparisons, while expanding research in lower- and middle-income countries is essential for addressing global variations in antibiotic use. In parallel, pharmacy practice would benefit from developing standardized, protocol-driven POCTs pathways, strengthening communication and patient education about antibiotic expectations, integrating adherence support into diagnostic services, and establishing sustainable remuneration and governance models. Building stronger connections with primary care and ensuring interoperability with referral pathways will also be critical for scaling these services safely and effectively.

Taken together, the findings of this review indicate that pharmacist-led POCTs holds significant promise for enhancing diagnostic-led triage and improving the appropriateness of antibiotic use. Realizing this potential will require a more coherent evidence base, greater methodological rigor, clearer system-level structures, and intentional integration of pharmacists into national antimicrobial stewardship strategies.

## 4. Methods

We conducted a systematic search in Google Scholar using the following combination of keywords: (“pharmacist” OR “community pharmacy”) AND (“rapid diagnostic test” OR “point-of-care test” OR “strep test” OR “influenza test” OR “UTI test”) AND (“antibiotic prescribing” OR “antimicrobial stewardship”). The search initially yielded 382 articles, including 210 published since 2021. Following title and abstract screening and full-text assessment using predefined inclusion and exclusion criteria, a total of 48 studies were included in the final review.

Google Scholar was selected intentionally because it provides the broadest coverage of interdisciplinary literature, including peer-reviewed clinical studies, public health publications, pharmacy practice research, policy papers, theses, and preprints. The topic of pharmacist-led point-of-care testing spans pharmacy, primary care, infectious disease, diagnostics, and health-policy, and much of this work—especially practice-based evaluations—is indexed inconsistently across conventional biomedical databases such as PubMed or Embase.

### 4.1. Screening Process

Two authors independently screened all titles and abstracts (n = 382). Articles that appeared potentially relevant were retrieved for full-text review. Disagreements were resolved by discussion until consensus was reached; a third author was available for adjudication but was not ultimately needed. Inclusion and exclusion criteria are presented in Table 2.

To increase transparency and reproducibility, we developed an algorithm summarizing the review workflow. The algorithm outlines the sequential steps of the review process, beginning with the Google Scholar search, followed by independent title and abstract screening, full-text assessment using the predefined inclusion and exclusion criteria, and final synthesis of the evidence. Incorporating this algorithm clarifies how studies were identified, selected, and analyzed, and visually demonstrates the decision-making pathway adopted by the review team.

### 4.2. Data Extraction

For all included articles, we extracted information on study type (clinical, policy, narrative review, etc.), POCTs examined, setting and population, antibiotic-related outcomes or insights, and identified gaps, barriers, or implementation challenges.

## 5. Conclusions

Pharmacist-led diagnostics are emerging as a promising component of antimicrobial stewardship, with growing policy support across multiple countries. Evidence to date suggests that POCTs integrated with structured pathways can reduce inappropriate antibiotic use, improve triage, and enhance access to care. However, the current evidence base is constrained by limited randomized trials, inconsistent outcome measures, scarce long-term follow-up, and substantial regulatory variation.

To inform safe and effective policy expansion, future research should prioritize multi-country cluster randomized trials, harmonised outcome metrics, cost-effectiveness analysis, and longitudinal monitoring of AMR indicators. Integrating adherence support and digital follow-up into pharmacy-based models represents a valuable next step. Strengthening the evidence underpinning pharmacist-led diagnostics will be essential for advancing AMS objectives and preserving antibiotic effectiveness globally.

## Figures and Tables

**Table 1:** Evidence from Clinical Studies on Pharmacist-Directed POCTs and Antibiotic Prescribing. Definition of abbreviations used in the table: CRP—C reactive protein, RCT—randomized controlled trial, LMIC—low- and middle-income countries.

Ref.	Type of Study	Country	Intervention	Result	Opportunities	Limitations
[5]	Parallel cluster RCT	Nigeria	- CRP POCTs kits; - Staff training for RTI management vs. usual care.	Reduced non-prescription antibiotic dispensing for RTIs.	- Feasible in LMIC pharmacies; may reduce inappropriate antibiotic use	- Limited to private pharmacies in one region - Resource-intensive (training, cost) - No long-term clinical outcomes
[3]	Cluster RCT with self-report behavioural questionnaire and process evaluation	UK	AMS behaviour change intervention: - training - TARGET TYI-RTI leaflets - Stewardship tools for pharmacy teams.	- Improved AMS behaviours and management processes - implied effect on reducing inappropriate antibiotic advice.	- Shows that AMS training and tools change pharmacy practice and advice patterns—scalable in well-resourced settings.	- Self-reported outcomes (behaviour), intermediate outcomes rather than direct measured prescribing rates - cluster size and generalizability.
[41]	Feasibility study/pilot	Australia	- CRP POCTs for RTI training for pharmacists	- Feasible and acceptable - CRP influenced patient perceptions and pharmacist advice, potentially reducing unnecessary GP visits/antibiotic expectations.	- Supports larger trials to measure effect on antibiotic prescribing.	- Small sample - Not randomized - Short follow-up - Outcome mainly feasibility and self-reported intention
[42]	Service pilot, qualitative evaluation	Australia	Implemented CRP service	Reduced perceived need for antibiotics.	- Practical implementation evidence - acceptability to pharmacists and potential to change advice/referral.	- Qualitative/service data only - No large-scale measurement of dispensing/prescribing changes.
[43]	Pilot feasibility evaluation	UK	- POC CRP testing in pharmacy - Linked with local doctors’ practices.	Feasible; suggested potential to reduce unnecessary doctor visits and to guide appropriate interventions.	Small-scale evidence that POC CRP in pharmacy can inform antibiotic decisions locally.	- Very small sample - Pilot, not controlled - Limited external validity.
[4]	Service evaluation/program evaluation (real-world roll-out)	Wales	- FeverPAIN/CENTOR screening	- Appropriate targeted antibiotic supply in pharmacy - Improved access and reduced general practitioners’ workload - Service expanded nationally after positive evaluation.	- Real-world model showing timely, targeted antibiotics	- Not randomized - Findings depend on service design and local regulatory/prescribing pathway - Limited long-term AMR outcomes.

**Table 2:** Inclusion and exclusion criteria of the literature.

Inclusion	Exclusion
Studies involving community pharmacists or community pharmacy practice. Articles addressing POCTs (e.g., rapid streptococcal, influenza, or UTI tests). Publications discussing or evaluating antibiotic prescribing, antimicrobial stewardship, or related decision-making. Primary studies, scoping reviews, systematic reviews, and narrative reviews that contributed relevant insights.	Studies conducted exclusively in hospital or non-pharmacy settings. Articles unrelated to antibiotic use or stewardship. Insufficiently detailed conference abstracts. Publications not available in English.

## Data Availability

The original contributions presented in this study are included in the article. Further inquiries can be directed to the corresponding author(s).
